# Integrative analysis of methylation and transcriptional profiles to predict aging and construct aging specific cross-tissue networks

**DOI:** 10.1186/s12918-016-0354-4

**Published:** 2016-12-23

**Authors:** Yin Wang, Tao Huang, Lu Xie, Lei Liu

**Affiliations:** 1 Shanghai Public Health Clinical Center and Institutes of Biomedical Sciences, Fudan University, Shanghai, China; 2 Shanghai Center for Bioinformation Technology, Shanghai Academy of Science and Technology, Shanghai, 201203 China; 3 Institute of Health Sciences, Shanghai Institutes for Biological Sciences, Chinese Academy of Sciences, Shanghai, 200031 People’s Republic of China

## Abstract

**Background:**

Aging is a complex process relating multi-scale omics data. Finding key age markers in normal tissues could help to provide reliable aging predictions in human. However, predicting age based on multi-omics data with both accuracy and informative biological function has not been performed systematically, thus relative cross-tissue analysis has not been investigated entirely, either.

**Results:**

Here we have developed an improved prediction pipeline, the Integrating and Stepwise Age-Prediction (ISAP) method, to regress age and find key aging markers effectively. Furthermore, we have performed a serious of network analyses, such as the PPI network, cross-tissue networks and pathway interaction networks.

**Conclusion:**

Our results find important coordinated aging patterns between different tissues. Both co-profiling and cross-pathway analyses identify more thorough functions of aging, and could help to find aging markers, pathways and relative aging disease researches.

**Electronic supplementary material:**

The online version of this article (doi:10.1186/s12918-016-0354-4) contains supplementary material, which is available to authorized users.

## Background

Aging is a multi-faceted and progressive bio-process for many organisms [1]. The aging process is composed by a serious of complex dynamic molecular interactions [2], which indicate key physiological phenotypes of human health. Moreover, dysfunctions of aging process have been shown to be involved in many disorders such as Parkinson disease [3], Alzheimers’ disease [4], many kinds of cancers [5] and so on. Therefore finding important aging markers could provide opportunities to predict healthy factors and improve diagnostic results for both pre- and pro- gnosis [6].

It has been reported that profiling patterns of crucial DNA methylation/mRNA markers change with the chronological age [7]. For example, many single tissue predictors (based on methylation or expression data) have been applied to identify aging biomarkers [8]. In addition, a multi-tissue predictor based on methylation data has been used to analyze aging functions [7]. Thus predicting age using multi-scale genome-wide data in normal tissues could provide reliable results of aging-related disease risks thereby [6]. As a result, integrating multi-omics data (i.e. epigenome and transcriptome data) with high predicting ability and meaningful biological results is required to analyze the aging process.

On the other hand, finding interactions between biomarkers is also important to identify characterizations of tissue/individual changes, such as phenotype stage and disease outcome [9, 10]. Reconstructing molecular networks also gives systematic approaches to deal with multi-scale data in aging analysis [11]. A previous study has constructed cross-tissue aging networks based on tissue-specific microarray mRNA data in mouse [11]. Nevertheless, integrative aging networks from multi-scale data (i.e. methylation and expression) have not been constructed entirely in Homo species.

In the present work, we developed a computational pipeline, the Integrating and Stepwise Age-Prediction (ISAP) method, to regress and predict age by integrating methylation and expression data in 9 normal tissues of human. The improved method found key integrative markers for both multi-tissue and tissue-specific models with high accuracy. Furthermore, a serious of network analyses such as the shortest Protein-Protein Interaction (PPI) network, cross-tissue co-profiling and pathway interaction networks revealed coordinated aging patterns in both multi-omics profiling and functional levels. The results showed integrative age-correlated profiles were associated with important pathway characteristics.

## Methods

### Data and pre-process

We obtained paired methylation, transcription and clinical data in 9 different normal tissues with more than 10 samples (BLCA: Bladder Urothelial tissue, BRCA: Breast invasive tissue, HNSC: Head/Neck squamous cell tissue, KIRC: Kidney renal clear cell tissue, KIRP: Kidney renal papillary cell tissue, LIHC: Liver tissue, LUAD: Lung tissue, PRAD: Prostate tissue, THCA: Thyroid tissue) from The Cancer Genome Atlas (TCGA, http://cancergenome.nih.gov) [12] platform (only using level-3 data). The age of each person came from TCGA clinical data. TCGA methylation data come from the Illumina Infinium Human Methylation27 BeadChip or the Illumina Infinium HumanMethylation450 BeadChip, therefore Illumina probe ID presented in both platforms were selected for further analysis [7]. Transcription data were obtained from RNASeq-v2 data (level-3). Both methylation and expression data were treated by a Singular Value Decomposition (SVD) [13] method (regress the first 3 principle components) to assess the sources of inter-sample variation separately in each tissue, and then were normalized to have zero mean and unit variance.

The choice of training data sets was guided by the following criteria the same as the previous study [7]: First, the training data should represent a wide spectrum of tissues and cell types; second, the mean age in the training data should be comparable to that of the test data. As a result, to predict age of each person in the multi-tissue model, 6 tissues (Bladder, Breast, Head/Neck, Kidney renal clear cell, Lung and Thyroid) were set as the training data (mean value ≈ 58 years). The rest 3 tissues (Kidney renal papillary cell, Liver and Prostate) were set as the independent test data (mean value ≈ 61 years). To train the multi-tissue model, each one out of the six tissues of training data was set as a set of temporary test data, so cross-validation of the multi-tissue model was performed as 6-fold. To train tissue-specific models, the common 5-fold cross-validation was performed for each tissue respectively.

### Lasso regression

Least absolute shrinkage and selection operator (Lasso) is a regression method performing both variable selection and regularization to improve the prediction accuracy and interpretability of the statistical model [14]. In this work Lasso was used to regress age using methylation data and the penalty parameter λ value was determined by cross-validation.

### Partial least-square (PLS) regression

The partial least-square regression (PLS) method is often used for dimension reduction when dealing with small-sized samples of gene expression data [15]. The algorithm is mainly performed as described by Höskuldsson [16].

In this work PLS was used to transformed high-dimension expression data before stepwise regression and the number of first modified direction vectors of PLS was finally determined (after stepwise regression) by cross-validation.

### Stepwise regression

The stepwise regression method is also widely used to select important features in regression problem in small-sample condition [17]. In this work the method was used to select important expression features (genes), and it works in the forward style as follows:

1. Sort gene expressions by their absolute correlation coefficients with the output in descending order. In this work the output was the residuals of ages from Lasso.
2. Add each sorted gene expression after PLS transformation individually. The number of selected genes was determined by cross-validation.

### Integrating and stepwise age-prediction method

In this paper, we presented an improved algorithm, the Integrating and Stepwise Age-Prediction (ISAP) method, to predict age by integrating Lasso, PLS and forward stepwise regression method based on paired methylation and expression data in normal tissues. Methylation data were used to regress age firstly, and then expression data were used to regress the residuals from methylation data. The improved computational method (ISAP) works as follows:

1. Predict age using Lasso regression based on methylation data, the penalty parameter λ value in Lasso is determined by cross-validation using training data, and save the residuals after Lasso regression.
2. Sort expression features (genes) by absolute correlation coefficients with the residuals in descending order.
3. Predict the residuals by forward stepwise regression method based on expression data. The expression data with selected features (genes) are transformed into full-ranked matrix by PLS firstly, and the number of selected genes are determined by cross-validation based on training data.
4. For selected expression features (genes), determined the number of first direction vectors of PLS by cross-validation based on the training data.
5. Caculate the final regression coefficients of aging markers (both methylation and expression), and save the regression coefficients.

**Fig. 1:**
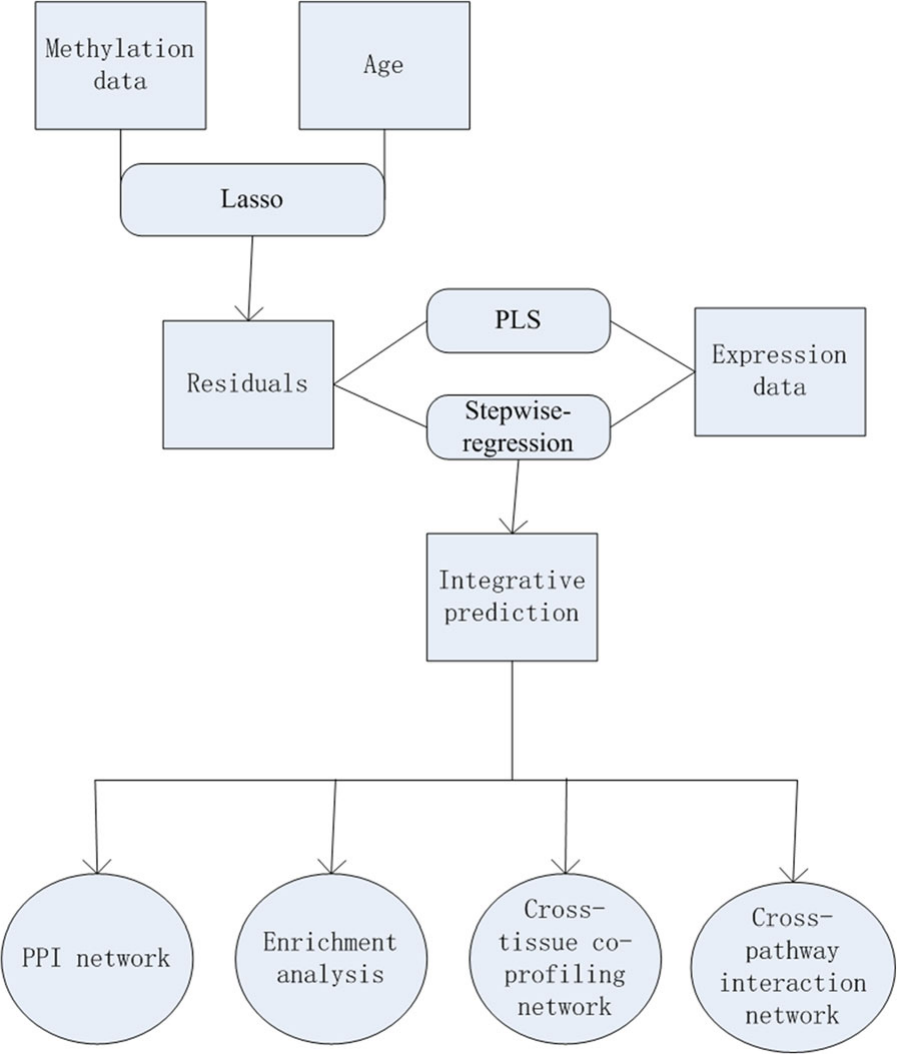
The computational pipeline (the ISAP method and further functional analyses) in this work

In this paper, both the multi-tissue model and tissue-specific models were calculated, and the selected aging markers were used to further functional analyses. The cross-validation was performed as 6-fold in the multi-tissue model, and 5-fold in tissue-specific models, separately. Age of both training data and test were subtracted mean value of training age before regression. The flowchart of ISAP and other succeeding works was shown in Fig. 1.

### Enrichment analysis

To gain understandings of gene sets statistically significantly associated with biological functions, enrichment analyses were carried out. Informations of Gene Ontology (GO) terms (including all GO gene sets), GO Biological Processes (BP), and KEGG pathways were downloaded from Gene Set Enrichment Analysis (GSEA) platform (http://software.broadinstitute.org/gsea/downloads.jsp, version 5.1) [18]. The hypergeometric test [19] was performed to estimate the enrichment of these selected markers compared to known terms/pathways. The formula of the hypergeometric test is:

1 $$ p\left(X\ge x\right)=1-{\displaystyle \sum_{k=0}^{x-1}\frac{C_M^k*{C}_{N-M}^{n-k}}{C_N^n}} $$

where *N* is the total gene number of the gene expression sets, *M* is the number of known genes sets (i.e. GO terms or KEGG pathway), *n* is the number of the candidate genes that we identified, and *k* is the number of common entries between them. *P* is the enrichment statistical significance of the test. Finally, the selected significantly enrichment *p*-values were controlled by False Discover Rate (FDR, <0.25) [18, 20].

### Protein-protein interaction (PPI) network

The background weighted PPI network was constructed using data from STRING database (http://string-db.org/, version 10) [21]. It weights protein-protein interactions by calculating confidence scores. In this work, 70% confidence score (>700) has been used as a cut-off for further analysis. Each pair of selected integrative markers was picked out and calculated their shortest pathway in PPI network using Dijkstra algorithm [22]. Finally the PPI network with shortest pathway among selected markers were constructed, and proteins/genes in the PPI network were sorted by their betweennesses in descending order.

To test whether the top betweenness genes were hubs in the background network or not, we ran a permutation to count the occurrence time of the top genes in the shortest paths between random selected genes (contained the same number of selected gene set of aging markers) when they have greater betweennesses than those in our study. We repeated this process 1000 times, and the *p*-value was calculated as the proportion of occurrence times of the top betweenness genes in 1000 permutations.

### Construction of integrative cross-tissue co-profiling network of aging

Age ≥ 60 was considered as ‘old’ age group, and age ≤ 50 was considered as ‘young’ age group. Tissues with more than 3 samples in both young and old group were selected to constructed cross-tissue network. As a result, 7 tissues were selected, they were: Breast invasive tissue, Head/Neck squamous cell tissue, Kidney renal clear cell tissue, Kidney renal papillary cell tissue, Liver tissue, Lung tissue, and Thyroid tissue. The number of tissue–tissue pairs was 21 in total.

For each tissue, profiles of selected aging markers (including both methylation and expression) were discretized using two thresholds mean+/-std. Then the discretized intertissue gene pairs (from 21 tissue-tissue pairs) within the same age group (young or old) were calculated the Kolmogorov-Smirnov (K-S) statistic [23]:

2 $$ K-S= \sup \left|{F}_1-{F}_2\right| $$

where *F*_*1*_ and *F*_*2*_ are the cumulative distribution of aging markers in one tissue and in another tissue, respectively. K-S values of both young age group and old age group were calculated separately. The absolute difference of K-S value between old and young group was set as the edge in the cross-tissue network, which would be filtered by a proper threshold (i.e. 0.95).

Moreover, all the aging markers in selected tissue-tissue pairs were enriched to GO terms by the hypergeometric test (FDR <0.25) to find functional characteristics of tissue-tissue cross-talk.

### Construction of integrative cross-tissue pathway interaction aging network

Selected aging markers in each tissue were enriched to KEGG pathway by the hypergeometric test using formula (1). Significant KEGG pathways (FDR <0.25) in each tissue (totally 7) was selected to further analysis. Three types of pathway interaction networks were considered: first, sum of absolute K-S value differences (> a moderate threshold, i.e. 0.6) was used as the connectivity of two pathways from two tissues; second, sum of all the absolute K-S value differences (with no thresholds) was used as the connectivity of two pathways between two tissues; third, sum of all the absolute K-S value differences (with a more rigorous KEGG enrichment FDR <0.1) was used as the connectivity.

## Results and discussion

### Aging regression results

To identify integrative aging biomarkers of multi-tissue, Lasso regression found 164 DNA methylation markers, and then the forward stepwise regression found 77 mRNA expression markers. Totally 241 aging markers were shown in Additional file 1 as well as their regression weights. Table 1 shows that the regression results of this computational pipeline, the ISAP method, get higher accuracy (with lower residual errors) compared to other common regression methods (i.e. Lasso, elastic net or PLS, either with methylation data or expression data).

**Table 1:** Regression results of ISAP and other methods

Method\residual errors	Training data	Test data
Lasso: methylation	120.3951	97.3057
Lasso: expression	148.3233	147.1687
Lasso: methylation and expression	130.6717	104.7382
PLS: methylation	120.1005	96.99
PLS: expression	187.9461	145.652
PLS: methylation and expression	130.8643	104.4602
elastic net: methylation	133.2711	94.871
elastic net: expression	141.8691	137.5191
elastic net: methylation and expression	129.1727	96.146
ISAP	116.3666	93.3048

Table 1 also shows that age prediction by methylation data with higher accuracy than using expression data. Moreover, simply combining methylation and expression could not improve regression results compared with using methylation data alone (shown in Table 1). Therefore, our improved computational method could integrate methylation and transcriptional data more effectively than other general methods.

**Fig. 2:**
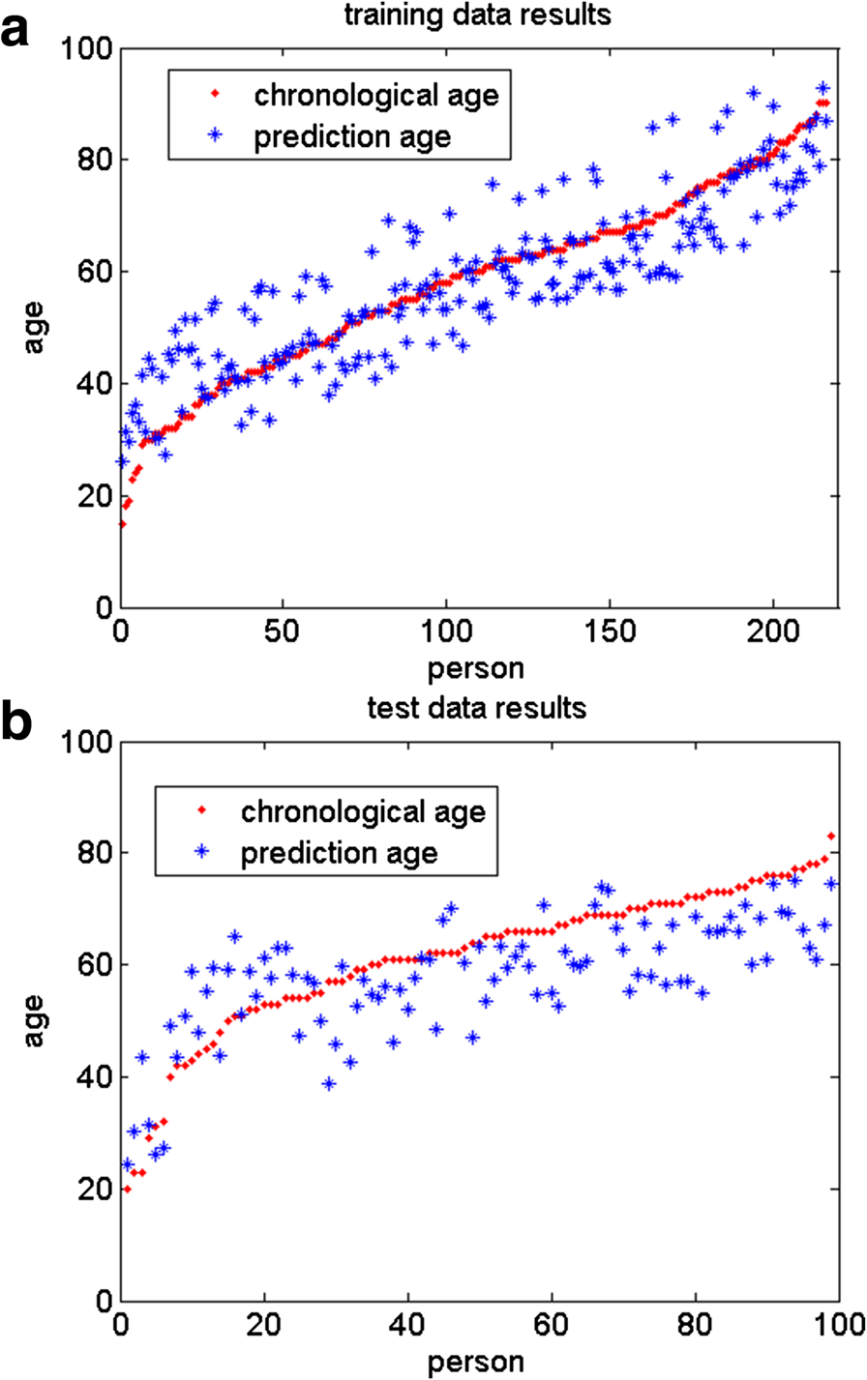
Regression results in the multi-tissue model. **a** Across all training data; **b** across all test data

**Fig. 3:**
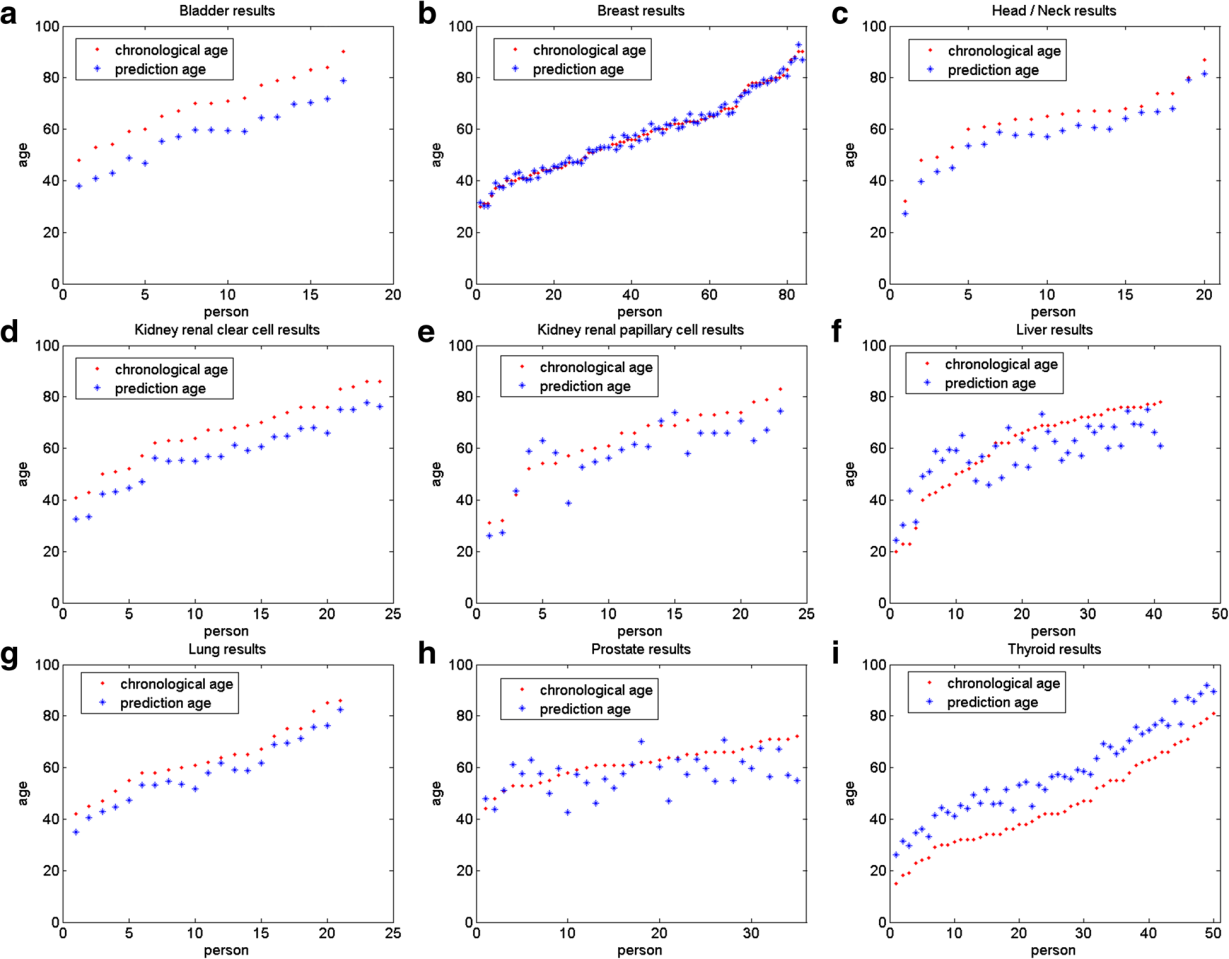
Regression results of each tissue in multi-tissue models. **a** Bladder Urothelial tissue; **b** Breast invasive tissue; **c** Head/Neck squamous cell tissue; **d** Kidney renal clear cell tissue **e** Kidney renal papillary cell tissue; **f** Liver tissue; **g** Lung tissue; **h** Prostate tissue; **i** Thyroid tissue. **a**-**d**, **g** and **i** are training data; **e**, **f** and **h** are test data

Figures 2 and 3 and Table 2 show more detailed (residual errors, Pearson correlation coefficients and median absolute difference errors) results in each tissue. The ISAP method not only got high accuracy on training tissues, but also predicted independent test tissues with enough accuracy (i.e. mean residual errors near 1.5 of each test tissue).

**Table 2:** Regression results in the multi-tissue model

Tissue	Residuals	Correlation	Median error	Samples	Type
Bladder	47.3902	0.9937	-11.0809	17	training
Breast	13.3672	0.9953	0.0509	84	training
Head/neck	26.9936	0.9892	-6.2175	20	training
Kidney renal clear cell	42.3265	0.9954	-8.3799	24	training
Lung	26.0847	0.9897	-5.2718	21	training
Thyroid	88.9724	0.9886	12.2297	50	training
Kidney renal papillary cell	39.4239	0.8735	-5.3395	23	test
Liver	63.6351	0.8111	-3.3107	41	test
Prostate	55.6967	0.3495	-3.836	35	test
training data	116.3666	0.8765	0.7133	216	
test data	93.3048	0.7413	4.5372	99	

Then we used our pipeline to regress age on each single tissue separately. Each identified integrative aging markers and their regression weights are also shown in Additional file 1. Table 3 shows regression results of tissue-specific biomarkers (details also shown in Additional file 1). Both multi-tissue and tissue-specific biomarkers were used to succeeding functional analyses (e.g. network construction).

**Table 3:** Regression results in tissue-specific models

Tissue	Residuals	Correlation	Median error	Samples	Methylation features	Expression features
Bladder	0.7736	0.9999	-0.0658	17	14	17
Breast	0.3187	1	0.0057	84	74	92
Head/neck	0.9342	0.9998	-0.0634	20	18	106
Kidney renal clear cell	3.1858	0.9987	0.0069	24	22	13
Kidney renal papillary cell	3.7071	0.9985	0.1355	23	22	4
Liver	10.0158	0.9953	0.1281	41	40	3
Lung	4.5721	0.9971	-0.038	21	19	14
Prostate	1.0266	0.9997	-0.014	35	34	38
Thyroid	17.8715	0.9892	-0.2026	50	48	16

### Functional/enrichment analysis and PPI-network

Firstly, selected integrative biomarkers were sorted by their absolute regression weights in descending order, which indicated their profiling patterns correlating the chronological age. For example, the marker with the greatest weight in the multi-tissue model was methylation profile of gene GPR45. Protein of GPR45 functioned in the central nervous system, and was reported to be related to aging significantly [24]. Moreover, the marker with the greastest weight in tissue-specific models was expression of gene CORO6 in the Kidney renal clear cell model, which has been reported that to be regulated by age [25].

Furthermore, we performed enrichment analysis of selected integrative biomarkers (multi-tissue and tissue-specific) on Biological Process (BP) of Gene Ontology (GO) and KEGG pathway using the hypergeometric test. In the multi-tissue model, the top GO biological process and KEGG pathway were positive regulation of immune system process (GO:0007059, *p*-value = 1.6643e-08, and FDR =1.3308e-05) and cell adhesion molecules (CAMs, *p*-value = 1.4205e-06, and FDR =2.4517e-04), respectively. It has been reported that inflammatory gene sets such as positive regulation of immune system process or dysfunction of immune system are induced by aging [26]. In addition, multiple changes in immune system occur and disrupt the regulation of body cells with aging in the immune dysregulation theory [26], which also coincides with our results. Many cell adhesion molecules have been indicated to be dependent on aging [27], too. In tissue-specific models, the top GO biological process and KEGG pathway were negative regulation of phosphate metabolic process (GO:0045936, *p*-value = 9.7695e-05, and FDR =0.0157) in kidney renal papillary cell and Antigen processing and presentation pathway (*p*-value = 3.3052e-06, and FDR =0.0006) in thyroid, respectively. Phosphate metabolic process has been reported to relate to aging diseases and cancer [28]. Moreover, autophagy is an important mechanism of intracellular pathogen’s antigens, and dysfuncition of autophagy is also regulated by aging [29]. As a result, the enrichment analyses indicated that functions of immune system related to aging deeply.

In addition, we constructed an undirected graph with the PPI data from STRING. Each pair of selected 241 integrative markers in the multi-tissue model were picked out and calculated their shortest path by Dijkstra algorithm. Then a sub-network composed of 161 out of the 241 markers was obtained based on shortest pathways, in which a total of 44244 protein-protein interactions of 2177 proteins annotating in the Ensemble Biomart database (http://ensembl.org/biomart/martview) were picked out. These genes/proteins were ranked by their betweennesses in descending order as well as permutation *p*-values (shown in Table 4). There were 7 out of the top 10 betweenness genes whose p-values were significant. Among these genes, TP53 has the largest betweenness of 1023 (permutation *p*-value = 0.016), meaning that there are 1023 shortest paths going through this gene. Accordingly, TP53 may play an important role in connecting the 161 candidate genes and hence may be related to aging. Obviously, TP53 is one of the most important key markers in cell cycle and cell apoptosis pathway, thus the cross-talk between TP53 and MTOR regulates cellular senescence, cancer and aging [30].

**Table 4:** Top aging markers with their betweennesses in the PPI network

Gene	Betweenness	*P*-value
TP53	1023	0.016*
HSP90AA1	665	0.009*
SRC	363	0.086
STAT3	263	0*
BMP2	254	0*
AKT1	243	0.759
CD8A	235	0*
EP300	229	0*
HSPA4	221	0*
IL6	207	0.018*

*: *p*-value < 0.05, significant

### Integrative aging-specific cross-tissue co-profiling networks

**Fig. 4:**
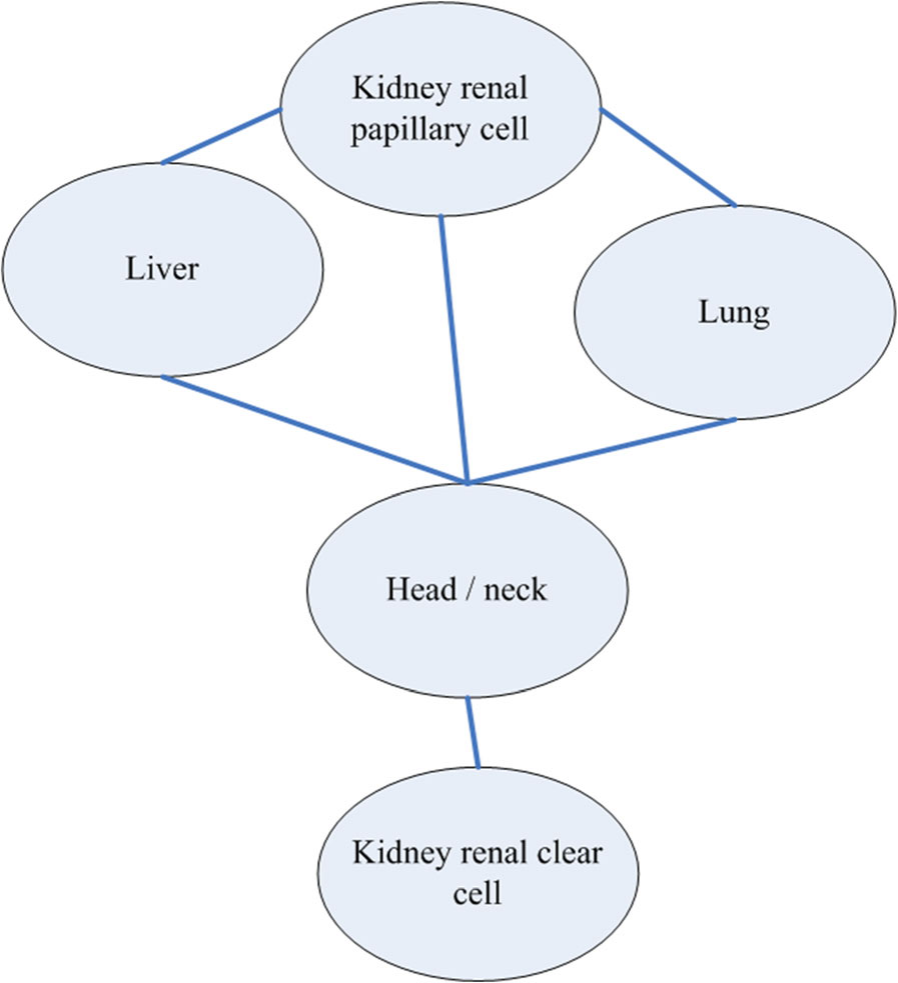
Patterns in the tissue–tissue pairs of aging

Aging is a complex process where many tissues and genes participate, thus gene profiling pattern pairs would be perturbed or regulated during aging. To investigate molecular aging profiling patterns of tissue-tissue pairs relating to different age group (young versus old people), cross-tissue co-profiling network were constructed using absolute difference of K-S statistics (>0.95). Finally 31 integrative inter-tissue pairs were obtained. The 31 molecular pairs belonged to 6 tissue-tissue pairs of aging, and they were shown in Fig. 4. Tissue of head/neck might be in the core status with the largest degree of 4 in the cross-tissue network, meaning that head/neck might affect other 4 tissues during the aging process.

Furthermore, each of the selected 31 pairs was calculated to investigate whether any pair shares the same GO term. As a result, the correlation of GATA4 in head/neck and EGFL7 in kidney renal clear cell shared most GO terms (number = 4), including system development (GO: 0048731), multicellular organismal development (GO: 0007275), anatomical structure development (GO: 0048856) and animal organ development (GO: 0048513). Since EGFL7 and GATA4 are related to aging [31, 32], it was possible the expression of EGFL7 in the kidney was regulated by the expression of GATA4 in head/neck, with this regulation changing with age. All the shared terms were related to cell/tissue developments, and tissue develpoments are correlated with aging [33], obviously.

In addition, all the aging specific genes with top high K-S differences (>0.85) were also performed enrichment analysis of GO terms by the hypergeometric test. The top GO terms were positive regulation of caspase activity (GO:0043280, *p*-value = 9.8594e-05, and FDR =0.0717). It is well known that caspase-dependent apoptotic signaling is vital to many human aging diseases [30, 34].

### Integrative aging-specific cross-tissue pathway interaction networks

**Fig. 5:**
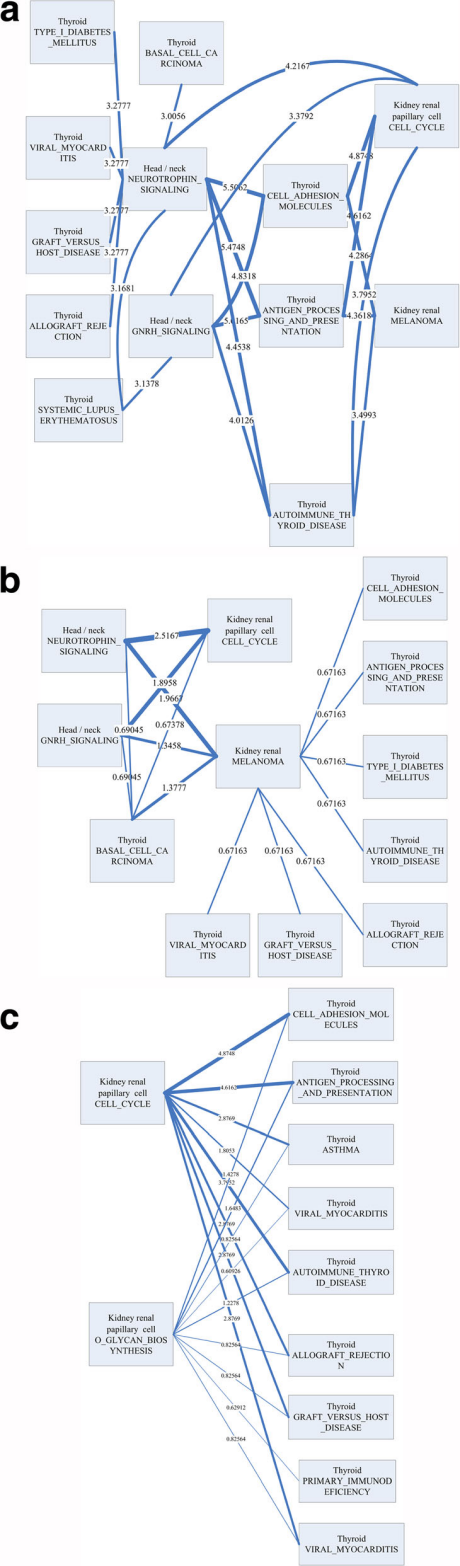
The cross-tissue pathway interaction networks of aging, and connectivities are shown in edges. **a** sum of absolute K-S differences (with a threshold >0.6) as connectivities; **b** sum of absolute K-S differences (with no thresholds) as connectivities; **c** sum of absolute K-S differences (FDR <0.1) as connectivities

To find inter-tissue pathway to pathway cross-talks, cross-tissue pathway interaction aging networks were constructed. Figure 5 depicts three types of pathway interaction cross-tissue networks. The first type showed the sum of absolute differences of K-S statistic with high difference (K-S difference >0.6). Figure 5a shows the pathway interaction networks of aging among head/neck, kidney and thyroid, and interaction between cell cycle and neurotrophin signaling pathway with the largest connectivity (=2.5167) might be important to aging. The second type showed the network based on sum of absolute differences of K-S statistic (only showed sum >3). Interaction between cell adhesion molecules and neurotrophin signaling pathway was with the largest connectivity (=5.5062, shown in Fig. 5b). The third type showed sum of absolute value of K-S statistics’ difference (FDR <0.1 as the threshold), and cross-talk between kidney and thyroid was the main pattern of the network. Figure 5c shows that interaction between cell cycle and cell adhesion molecules might be critical to aging (connectivity =4.8748).

In immunosenescence theories of aging, either innate or adaptive immune responses are related to the aging process [26]. For instance, cellular senescence is believed to be involved immune and aging progresses, and suppressed relative pathways such cell cycle, pathway of cancers and so on [26, 30, 35]. Cell adhesion cascades appear to affect the functional capacity of cells during aging [27, 36]. Moreover, both cell adhesion and neurotrophin cooperatively perform critical functions in the aging process responding to the immune system [37, 38]. Our results found that the cross-talk among key pathways (i.e. cell cycle, cell adhesion and neurotrophin signaling) played important roles in the aging process altogether. Furthermore, head/neck and kidney might be in core status to regulate the aging process and relative pathways in other tissues.

## Conclusion

Predicting age in human normal tissues is fundamental to aging researches. In this paper, we developed an improved method, the ISAP pipeline, to integrate both methylation and expression data for age prediction. The ISAP method predicted age more accurately than other popular methods. Furthermore, the PPI network and enrichment analyses also find core aging genes and pathways.

In addition, network analysis could also help to identify aging related genes/pathways between different tissues. We have performed a serious of network analyses of aging specific markers, and find important profiling patterns and pathway interactions. Our results confirmed existing aging theories or hypotheses and could improve further aging researches.

## Additional file

Additional file 1: Integrative age predictors and their weights. Note: type 1 indicates methylation data, and type 0 indicates expression data; tissue with 0 indicates the multi-tissue model, and 1-9 indicate each of 9 normal tissues (Bladder Urothelial tissue, Breast invasive tissue, Head/Neck squamous cell tissue, Kidney renal clear cell tissue, Kidney renal papillary cell tissue, Liver tissue, Lung tissue, Prostate tissue, Thyroid tissue), separately. (XLS 97 kb)
